# LRP1 expression in microglia is protective during CNS autoimmunity

**DOI:** 10.1186/s40478-016-0343-2

**Published:** 2016-07-11

**Authors:** Tzu-Ying Chuang, Yong Guo, Scott M. Seki, Abagail M. Rosen, David M. Johanson, James W. Mandell, Claudia F. Lucchinetti, Alban Gaultier

**Affiliations:** Center for Brain Immunology and Glia, Department of Neuroscience, University of Virginia, Charlottesville, VA USA; Graduate Program in Pathology, University of Virginia, Charlottesville, VA USA; Medical Scientist Training Program, University of Virginia, Charlottesville, VA USA; Department of Neurology, Mayo Clinic, Rochester, MN USA; Department of Pathology, University of Virginia, Charlottesville, VA USA

**Keywords:** Microglia, Inflammation, LRP1, NF-kB, Multiple sclerosis

## Abstract

Multiple sclerosis is a devastating neurological disorder characterized by the autoimmune destruction of the central nervous system myelin. While T cells are known orchestrators of the immune response leading to MS pathology, the precise contribution of CNS resident and peripheral infiltrating myeloid cells is less well described. Here, we explore the myeloid cell function of Low-density lipoprotein receptor-related protein-1 (LRP1), a scavenger receptor involved in myelin clearance and the inflammatory response, in the context of Multiple sclerosis. Supporting its central role in Multiple sclerosis pathology, we find that LRP1 expression is increased in Multiple sclerosis lesions in comparison to the surrounding healthy tissue. Using two genetic mouse models, we show that deletion of LRP1 in microglia, but not in peripheral macrophages, negatively impacts the progression of experimental autoimmune encephalomyelitis, an animal model of Multiple sclerosis. We further show that the increased disease severity in experimental autoimmune encephalomyelitis is not due to haplodeficiency of the *Cx3cr1* locus. At the cellular level, microglia lacking LRP1 adopt a pro-inflammatory phenotype characterized by amoeboid morphology and increased production of the inflammatory mediator TNF-α. We also show that LRP1 functions as a robust inhibitor of NF-kB activation in myeloid cells via a MyD88 dependent pathway, potentially explaining the increase in disease severity observed in mice lacking LRP1 expression in microglia. Taken together, our data suggest that the function of LRP1 in microglia is to keep these cells in an anti-inflammatory and neuroprotective status during inflammatory insult, including experimental autoimmune encephalomyelitis and potentially in Multiple sclerosis.

**Electronic supplementary material:**

The online version of this article (doi:10.1186/s40478-016-0343-2) contains supplementary material, which is available to authorized users.

## Introduction

Multiple sclerosis (MS) is an inflammatory autoimmune disease characterized by the destruction of myelin in the central nervous system (CNS) and irreversible neurodegeneration [1]. Myelin destruction is initiated by the myelin-reactive T cells, and is further amplified by the inflammatory response of myeloid cells, including the brain resident microglia and the brain infiltrating inflammatory macrophages [2–6].

Although it is clear that microglia and macrophages participate in the MS pathology development, their precise functions remain extremely controversial [7]. On one hand, microglia are proposed to be protective and relatively immune silent in MS, when compared to macrophages [8], while other studies demonstrate that microglia themselves can contribute to disease initiation. Indeed, mice with a microglia-specific deficiency in the activation of the transcriptional master regulator of inflammation, NF-kB, are protected from EAE [9].

Low-density lipoprotein receptor-related protein-1 (LRP1), or CD91, is a scavenger receptor involved in the removal of myelin debris, as well as necrotic and apoptotic cells [10–12]. The clearance of myelin debris generated during demyelination episodes is critical for the regenerative capacity of the CNS. Improper myelin debris clearance by microglia has been shown to delay recovery in a mouse model of demyelination [13]. Besides mediating the removal of cellular debris, LRP1 can also directly influence cellular signaling pathways [14]. We have previously shown that LRP1 functions as a broad inhibitor of NF-kB activity and inflammatory mediator production [15]. Because inhibition of NF-kB is known to offer protection during experimental autoimmune encephalomyelitis (EAE), a mouse model of MS [16–20], placement of LRP1 at the crossroads of inflammation and phagocytosis raises the possibility of its involvement as a central regulator of MS pathology.

In this study, we explore the precise contribution of LRP1 during MS, using EAE as an animal model. We show that LRP1 protein expression is increased in the myeloid and astrocytic compartments during active disease in MS, in comparison to the healthy CNS. To elucidate the role of LRP1 in myeloid cells, we induced EAE in mice lacking LRP1 in either the peripheral or resident myeloid cells. To our surprise, deletion of LRP1 in peripheral macrophages had no effect on the disease severity, whereas in mice lacking LRP1 specifically in microglia, disease was intensified, suggesting a protective role for LRP1 in microglia. Further experiments revealed that, at baseline, microglia deficient in LRP1 adopt a pro-inflammatory phenotype and exhibit morphological changes that parallel those observed in microglia under inflammatory conditions. On a mechanistic level, our results suggest that the increased pathology in mice with LRP1 deficient microglia originates from uncontrolled NF-kB activity. Taken together, our results demonstrate that LRP1 has a protective role in microglia and that the alteration of their function can drastically impact EAE outcome.

## Materials and methods

### Human samples

The human pathology study was approved by the Institutional Review Board of Mayo Clinic, Rochester, MN. Six clinically and pathologically confirmed multiple sclerosis autopsy cases were used in this study. Formalin-fixed paraffin-embedded 5 μm thick sections were stained for H&E, Luxol fast blue and periodic acid Schiff (LFB/PAS), and Bielschowsky’s silver stain for routine pathology analysis. Immunohistochemistry was performed with the avidin–biotin-complex method according to the manufacturer’s instructions (Vectorlab). The following primary antibodies are used: LRP1 (1:200, Abcam) myelin proteolipid protein (PLP, 1:500, Serotec), myelin-associated glycoprotein (MAG, 1:1000, Abcam), myelin oligodendrocyte glycoprotein (MOG, 1:1000, Abcam), CD68 (1:100, DAKO), and glial fibrillary acidic protein (GFAP, 1:100, DAKO). Steamed antigen retrieval with citrate buffer (pH 6.0) was performed for LRP1, MAG, MOG, CD68, and GFAP. The demyelinating activity in the parenchyma for each block was evaluated in regions derived from the 6 cases, according to the myelin debris in macrophages as previously described [21].

### Mice

C57BL/6 mice with loxP sites flanking the LRP1 gene [22] were crossed with *Cx3cr1*^cre^, *Cx3cr1*^creER^ [9] and *LysM*^cre^ [23] mice to generate *LRP1*^fl/fl^*Cx3cr1*^cre^, *LRP1*^fl/fl^*Cx3cr1*^creER^ and *LRP1*^fl/fl^*LysM*^cre^ mice. To induce Cre recombinase activity, 4 to 6-weeks old *LRP1*^fl/fl^*Cx3cr1*^creER^ mice were injected twice i.p. with tamoxifen (250 mg/kg in corn oil, Sigma) 1 week apart as described in the literature [9]. For controls, littermates carrying loxP-flanked LRP1 alleles without Cre recombinase expression or age and sex matched mice with *Cx3cr1*^creER^ activity without loxP-flanked LRP1 alleles, were used. All animal procedures were approved by the University of Virginia’s Animal Care & Use Committee.

### Experimental autoimmune encephalomyelitis

EAE was induced in female mice (8 to 12 weeks) by subcutaneous injection of MOG_35-55_ peptide (100 μg, CSBio) emulsified in complete Freund’s adjuvant containing *Mycobacterium tuberculosis* (1 mg/ml, BD). Pertussis toxin (200 ng, List Biologicals) was administered i.p. on the day of and 1d after MOG immunization. For experiments with *Cx3cr1*^creER^ mice, EAE was induced 4 weeks after the tamoxifen treatment as previously described [9]. For clinical evaluation, mice were scored daily: 0-no clinical disease, 1-limp tail, 2-hindlimb weakness, 3-hindlimb paralysis, 4-partial front limb paralysis, 5-moribund.

### Antigen recall assay

Antigen recall assay was performed as previously described [9]. Briefly, single cell suspensions were prepared from draining inguinal lymph nodes of Cx3cr1^creER^*Lrp1*^fl/fl^ and control mice 7 days post immunization with MOG_35-55_ peptide. Cells were treated with various concentrations of MOG_35-55_ peptide as indicated in the manuscript for 24 h. ELISA was used to measure IFN-γ and IL-17A production. To determine proliferation, BrdU (10 μM, BD) was added to the culture media and BrdU incorporation was measured 16 h after treatment by flow cytometry.

### Blood brain barrier assay

Six hours prior to the assay, mice were injected i.p. with LPS (6 mg/kg). Heparin (20 U/mouse) was administered i.p. and followed, 30 min later, by 2 % sodium fluorescein (200 μl, NaF). After another 30 min, the mice were perfused, the meninges removed, and the brains weighed and isolated in 50 % trichloroacetic acid overnight at 4 °C. The tissue was finely diced and homogenized. After centrifugation (13,000 x g, 10 min, 4 °C) and neutralization of the acid with NaOH (5 M), 200 μl of the clarified homogenate was plated in 96 well black/clear bottom plates and fluorescence measured. Data is expressed as amount of tracer per gram of tissue.

### Cell isolation from the CNS

Brain and/or spinal cords were removed and digested in HBSS containing collagenase IV (2 mg/ml) and DNase (20 U/ml) for 45 min at 37 °C. Cells were isolated using a 30 %/70 % Percoll gradient (GE Healthcare) [24]. For adult primary microglia culture, cells were positively selected using CD11b magnetic microbeads (Miltenyi Biotec) after digestion. For flow cytometry analysis, single cell suspensions were stained with antibody against CD45 (30-F11, eBioscience), CD11b (M1/70, eBioscience) and LRP1 (ab92544, 1:1000, Abcam). Flow cytometry analyses were performed on a 10-color Beckman Coulter Gallios flow cytometer and data were analyzed with FlowJo software (TreeStar).

### Immunofluorescence

Anesthetized mice were perfused transcardially with PBS, followed by 4 % paraformaldehyde (PFA) prepared in PBS. Tissues were post-fixed for at least 24 h in 4 % PFA, and transferred to 20 % sucrose solution before sectioning. Cryosections (40 μm) were permeabilized in 0.5 % PBS-Tween for 15 min and washed twice with PBS. After blocking in 5 % serum in PBS for 2 h at room temperature, sections were incubated overnight at 4 °C with antibody against Iba1 (ab5076, 1:400, Abcam), LRP1 (ab92544, 1:250, Abcam) and CD68 (14-0688, 1:50, eBioscience). After washing, sections were incubated with secondary antibodies conjugated to Alexa488 or Alexa647 (Life Technologies). Sections were mounted with Prolong Gold Anti-Fade Reagent containing DAPI (Life Technologies). All sections were imaged with a Leica TCS SP8 confocal microscope and analyzed with ImageJ. Sholl analysis was performed as described [24].

### Immunohistochemistry

Slides were deparaffinized using xylenes and an ethanol gradient. Heat induced antigen retrieval was performed for 10 min (Sodium Citrate Buffer, pH 6). Staining for CD68, Iba1, and LRP1 was conducted using ImmPress reagents according to manufacturer’s instructions (Vector lab). Adjacent sections were stained with LFB for demyelinating plaques. Histological analysis was performed by two independent investigators, blinded to the status of LRP1.

### Preparation of Bone Marrow Derived Macrophages

Mice were euthanized and dissected to retrieve femurs and tibias. BMDM cells were cultured at 37 **°**C 5 % CO_2_ in high glucose DMEM with L-glutamine (GE Life Sciences, SH30022) supplemented with 10 % fetal bovine serum (Atlanta Biologicals, s12450), sodium pyruvate (Life Technologies, 11360-070), 1x Penicillin-Streptomycin (Life Technologies, 151490-122) and 30 % L929-conditioned media. Fresh media was supplemented at day 4, and the cells were replated on day 6. In some experiments, BMDM were treated with LPS (Sigma) and other TLR ligands (InvivoGen) as described in the text.

### ELISA

ELISA analyses for IL-6 and TNF-α were performed as previously described [25]. Antibodies used were: anti-mouse IL-6 (MP5-20 F3, Biolegend) 0.5 μg/mL; biotin anti-mouse IL-6 (MP5-32C11, Biolegend) 1 μg/mL; anti-mouse TNF-α (R&D systems, AF-410-NA) 0.5 μg/mL; biotin anti-mouse TNF-α (R&D systems, BAF410) 0.25 μg/mL.

### Immunoblot

Proteins were extracted in RIPA buffer supplemented with protease inhibitor cocktail (Roche) and the phosphatase inhibitor sodium orthovanadate (2 mM). After incubation for 15 min on ice, the lysates were centrifuged (13,000 x g, 4 °C) and protein quantified using BCA assay. Protein samples were run on a Protean TGX gel (Bio-Rad) and transferred to a PVDF membrane. After blocking with 5 % milk in TBS-Tween for 1 h at room temperature, membranes were incubated overnight with primary antibody. After washing, membranes were incubated with HRP conjugated secondary antibodies (Pierce) for 1 h at room temperature and developed with Western Lighting Plus ECL (Perkin Elmer).

### Quantitative PCR

RNA was extracted using the Isolate II Kit (Bioline) according to manufacturer’s instructions and cDNA prepared with the iScript cDNA synthesis kit (Bio-Rad). qPCR was conducted with TaqMan primers (Applied Biosystems) for LRP1, IL-6, TNF-α, Il-1β, and normalized to GAPDH. Reactions were run on a PikoReal PCR system (Thermo Fisher) with the 2x NO-ROX Sensifast Mix or 2x SYBR Sensifast Mix (Bioline).

## Results

### LRP1 expression is increased in MS lesions

**Fig. 1:**
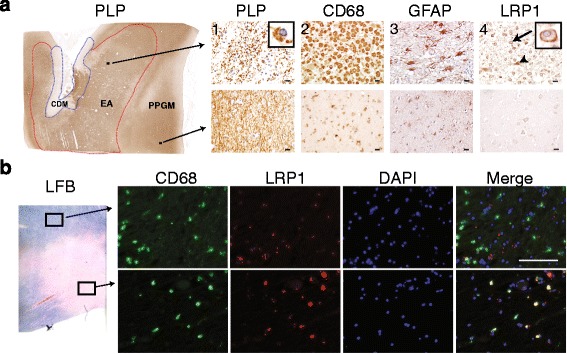
LRP1 expression is increased in MS lesions. **a** Immunohistochemistry on consecutive sections from an early active (EA) MS lesion (upper row) shows: (1) myelin (PLP) laden macrophages consistent with ongoing demyelinating activity, (2) abundant macrophage infiltration (CD68), (3) hypertrophic reactive astrocytes indicating gliosis (GFAP), and (4) LRP1 immunoreactivity present on both astrocytes (arrowhead) and macrophages (arrow and inset). In contrast, the periplaque gray matter (PPGM, lower row) shows: (1) normal appearing myelin (PLP), (2) limited microglial reactivity (CD68), (3) astrocytes with regular size and morphology (GFAP), and (4) limited LRP1 immunoreactivity. (Scale bars = 20 μm). **b** Luxol Fast Blue histology (LFB) and immunofluorescence for CD68 and LRP1 shows that myeloid cells express LRP1 within the lesion (Scale bar = 100 μm)

Our previous studies demonstrated that LRP1 functions as a receptor for myelin phagocytosis and a broad inhibitor of inflammation [11, 15], two key functions linked with MS pathogenesis. Because we observed increased expression of LRP1 in the CNS during an animal model of MS, experimental autoimmune encephalomyelitis (EAE) [11], we decided to probe the potential function of LRP1 during the human disease. We began by examining LRP1 expression in MS lesions at different stages of demyelinating activity. In the normal appearing periplaque gray matter, LRP1 was mainly detected in neurons (Fig. 1a, *lower row*), in agreement with a previous publication [26]. However, within the early active lesions, characterized by the presence of CD68+ myeloid cells containing myelin debris and GFAP+ reactive astrocytes, LRP1 expression was increased (Fig. 1a, *upper row*). Morphological analysis suggested that LRP1 expression was most prominent in myeloid cells (arrow) and astrocytes (arrowhead) in the early active lesion. The results from a total of 6 MS autopsies are summarized in Table 1. To confirm that myeloid cells express LRP1 within the active lesion, we performed double immunofluorescence with antibodies against LRP1 and CD68. Within the lesion, CD68+ cells are also LRP1+, while LRP1 is not detectable within the myeloid cells outside the lesion, confirming our immunohistochemistry results (Fig. 1b). LRP1 immunoreactivity in myeloid cells was also not present in inactive lesions (Table 1). Taken together, our results demonstrate that LRP1 expression in myeloid cells and astrocytes is increased during MS pathology.

**Table 1:** LRP1 expression in MS: 6 clinical and pathologically confirmed multiple sclerosis autopsy samples were analyzed

Lesion stage from MS autopsy	LRP1 expression in the Lesion		
	Myeloid Cells	Neurons	Astrocytes
Early Active	100 % (2/2)	NA^a^ (0/2)	100 % (2/2)
Late Active	0 % (0/1)	NA^a^ (0/1)	100 % (1/1)
Periplaque White Matter	0 % (0/5)	NA^a^ (0/5)	100 % (5/5)
Non-Affected White Matter	0 % (0/5)	NA^a^ (0/5)	100 % (5/5)
Chronic Demyelination	0 % (0/4)	100 % (4/4)	100 % (4/4)
Non-Affected Gray Matter	0 % (0/5)	100 % (5/5)	100 % (5/5)

Formalin-fixed paraffin-embedded 5 μm thick sections were stained for H&E, Luxol fast blue and periodic acid Schiff (LFB/PAS), and Bielschowsky’s silver stain for routine pathology analysis. For cellular analysis, slides were stained for LRP1, myeloid marker CD68, and reactive astrocyte marker GFAP. Neurons were not detected in white matter

^a^ *NA* not applicable

### Microglial LRP1 is protective during experimental autoimmune encephalomyelitis

**Fig. 2:**
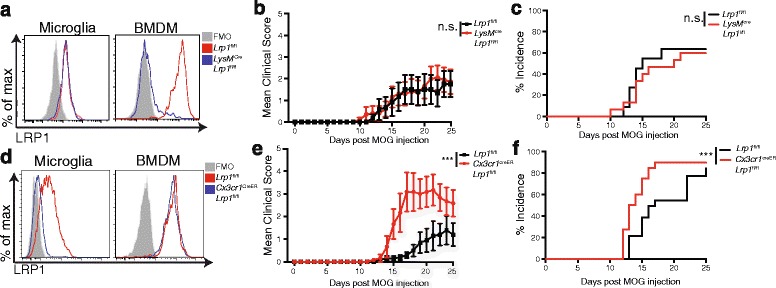
Deletion of LRP1 from microglia, but not from peripheral myeloid cells, exacerbates EAE progression. **a** LRP1 expression was determined by flow cytometry on microglia and BMDM isolated from *LysM* ^cre^-*Lrp1* ^fl/fl^ and *Lrp1* ^fl/fl^ mice. **b** Clinical scores and **c** incidence of *LysM* ^cre^-*Lrp1* ^fl/fl^ and *Lrp1* ^fl/fl^ mice (*n* = 7–9 for each group, representative of 3 independent experiments). **d** LRP1 expression was determined by flow cytometry on microglia and BMDM isolated from *Cx3cr1* ^creER^-*Lrp1* ^fl/fl^ and *Lrp1* ^fl/fl^ mice. **e** Clinical scores and **f** incidence of *Cx3cr1* ^creER^-*Lrp1* ^fl/fl^ and *Lrp1* ^fl/fl^ mice (*n* = 6–10 for each group, representative of 3 independent experiments.). 2-way ANOVA was used for the mean clinical score and the log-rank (Mantle-Cox) test was used for incidence. ***p < 0.001, mean ± s.e.m

Myeloid cells involved in MS pathology are from two different origins, the yolk sac derived CNS resident microglia [27] and the peripheral infiltrating macrophages. To explore the function of myeloid LRP1, we used the well-accepted animal model of MS, EAE, in two different mice strains lacking LRP1 either in peripheral myeloid cells or in microglia. To study the function of LRP1 in peripheral myeloid cells, we induced EAE in LRP1 deleted *LysM*^cre^-*Lrp1*^fl/fl^ or the control *Lrp1*^fl/fl^ mice with the myelin oligodendrocyte glycoprotein (MOG_35-55_) peptide and scored the mice daily. While LysM is highly expressed in peripheral myeloid inflammatory cells, LysM expression in microglia is variable [9, 28]. Using *LysM*^cre^-*Lrp1*^fl/fl^ mice [29], we observed complete deletion of LRP1 in bone marrow derived macrophages (BMDM), while we observed little to no decrease in basal microglial LRP1 expression, as demonstrated by flow cytometry (Fig. 2a). Given that we have previously shown that macrophage LRP1 functions as an inhibitor of inflammation, we were surprised to find that deletion of LRP1 in peripheral myeloid cells in *LysM*^cre^-*Lrp1*^fl/fl^ mice had no effect on the clinical score or incidence of EAE (Fig. 2b-c). Clinical scores were also similar when the amount of mycobacterium was increased to induce a stronger EAE disease course (Additional file 1: Figure S1) [30].

Next, we generated a new mouse strain to study the role of LRP1 in microglia during EAE. The *Lrp1*^fl/fl^ strain [22] was crossed to the recently developed *Cx3cr1*^creER^ strain [31] to generate *Cx3cr1*^creER^-*Lrp1*^fl/fl^ animals. Because CX3CR1 is expressed in microglia and monocytes [9], tamoxifen treatment initially leads to the deletion of LRP1 in both cell types. However, the pool of circulating monocytes that can give rise to macrophages during inflammation is continuously renewed by progenitors from the bone marrow, while the long-lived brain resident microglia are not replenished and remain LRP1 deleted [2, 9]. Indeed, 1 month after the administration of tamoxifen, we could still detect deletion of LRP1 in microglia, while LRP1 expression in bone marrow derived macrophages was comparable to control cells (Fig. 2d). EAE was induced by immunization with MOG_35-55_ peptide. LRP1 deletion in microglia results in a significant increase in clinical scores and overall incidence of EAE (Fig. 2e-f). Difference in the clinical score was not due to CX3CR1 haplodeficiency, as induction of EAE in animals that were not treated with tamoxifen showed no significant difference between groups (Additional file 1: Figure S2). Our results suggest that LRP1 expression in microglia has a protective role in EAE, significantly reducing the disease severity.

#### Demyelination and peripheral immune cell recruitment are elevated in mice lacking LRP1 in microglia

**Fig. 3:**
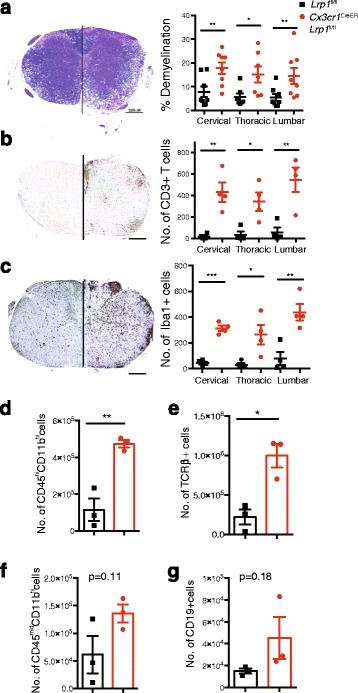
Microglial LRP1 deficient mice have increased demyelination and numbers of immune cells in the CNS during EAE. Spinal cord sections of *Cx3cr1* ^creER^-*Lrp1* ^fl/fl^ and *Lrp1* ^fl/fl^ mice were stained with Luxol Fast Blue **a** at day 30 (*n* = 4 for each group), and CD3 **b** (*n* = 4 for each group) and Iba1 **c** (*n* = 4 for each group) at day 15. Cells were manually quantified. For LFB, 2 sections per spinal cord level are plotted from each animal. For CD3 and Iba1, each point represents one animal. Immune cells were isolated by percoll gradient from spinal cords of *Cx3cr1* ^creER^-*Lrp1* ^fl/fl^ and *Lrp1* ^fl/fl^ mice. Number of **d** peripheral myeloid cells (CD11b^hi^CD45^hi^), **e** T cells (TCRβ+) cells, **f** microglia (CD11b^hi^CD45^mid^), and **g** B cells (CD19+) were determined by flow cytometry analysis (*n* = 3 for each group). *p < 0.05, **p < 0.01; Student’s t-test, mean ± s.e.m

To explore the pathological conditions leading to an exacerbated disease course in EAE mice lacking LRP1 expression in microglia, we performed histological analysis of the spinal cord during the chronic phase of the disease (day 30). Myelin staining of the spinal cord sections with Luxol Fast Blue revealed that *Cx3cr1*^creER^-*Lrp1*^fl/fl^ mice have significantly more white matter demyelination than the control group (Fig. 3a). This difference was statistically significant at all of the spinal cord levels that were examined: Lumbar, thoracic and cervical levels. Immune cells are responsible for driving demyelination during EAE. To explore if LRP1 deficiency in microglia could alter immune cell numbers, we performed flow cytometry and immunohistochemical analysis of the spinal cords at the onset of disease (day 15). Histologically, we find increased numbers of CD3+ T cells and Iba1+ cells in the *Cx3cr1*^creER^-*Lrp1*^fl/fl^ mice at disease onset (Fig. 3b-c), especially evident in the dense infiltrates in the white matter of the spinal cord. By flow cytometry (Additional file 1: Figure S3), our results reveal that the number of T cells and peripheral myeloid (CD11b^hi^CD45^hi^) cells was significantly increased in animals lacking LRP1 (Fig. 3d-e). Finally, there was a trend towards increased numbers of microglia (CD11b^mid^CD45^hi^) and B cells (CD19^+^) (Fig. 3f-g). Taken together, our results show that mice lacking LRP1 in microglia had increased EAE susceptibility that is associated with robust demyelination and increased infiltration of immune cells from the innate and adaptive arms of the immune system.

#### Lymphocyte activation is not affected in the absence of LRP1

**Fig. 4:**
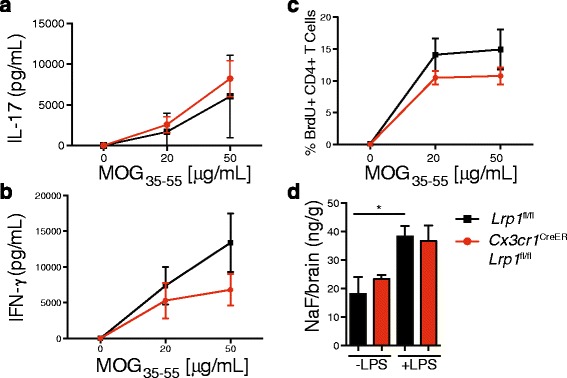
Immune response and blood brain barrier permeability are not altered in microglial LRP1 deficient mice. Seven days after MOG immunization, draining lymph nodes were isolated from *Cx3cr1* ^creER^-*Lrp1* ^fl/fl^ and *Lrp1* ^fl/fl^ mice and incubated with MOG. **a**-**b** IFN-γ and IL-17A production were determined by ELISA after 24 h. **c** BrdU incorporation by T cells was determined after 16 h by flow cytometry (*n* = 3–5 mice were used per group per experiment). Statistical analysis was performed with 2-way ANOVA. **d** The amount of fluorescein present in brains following LPS treatment (6 mg/kg) was measured in *Cx3cr1* ^creER^-*Lrp1* ^fl/fl^ and *Lrp1* ^fl/fl^ mice to assess function of the blood brain barrier. Statistical analysis was performed with Student’s t-Test; means ± SEM

LRP1 is a multifunctional receptor with roles that could impact EAE progression at different levels [14]. As LRP1 functions in antigen presentation [32], and CX3CR1 may be expressed in subsets of antigen presenting cells (APC) in the periphery [33], we investigated the antigen recall response of inguinal lymph node T cells in the *Cx3cr1*^creER^-*Lrp1*^fl/fl^ mice 7 days after immunization. Treatment with MOG_35-55_ peptide did not reveal any significant differences in T cell production of IFN-γ and IL-17A, as determined by ELISA (Fig. 4a-b). Furthermore, T cell proliferation measured by BrdU incorporation was also comparable to control cells (Fig. 4c). Taken together, these data suggest that increased disease severity in EAE for *Cx3cr1*^creER^-*Lrp1*^fl/fl^ mice is not linked to impaired antigen presentation in the periphery.

#### Function of the blood brain barrier is intact in mice lacking microglial LRP1

Blood Brain Barrier (BBB) permeability disruption is an essential event in providing access to peripheral lymphocyte infiltration into the CNS during EAE [34], and microglia are known to modulate the function of the BBB [35]. To investigate if LRP1 deletion in microglia can impact BBB permeability, we induced microgliosis in *Cx3cr1*^creER^-*Lrp1*^fl/fl^ and control mice by injecting LPS, as previously described [36]. LPS administration leads to increased extravasation of fluorescein in comparison to the basal level, as expected [35]. However, BBB permeability was not affected by the status of LRP1 expression in microglia (Fig. 4d). These results suggest that LRP1 function in microglia is likely not critical for the maintenance of the BBB function.

#### Microglial morphology is altered by LRP1 deficiency

**Fig. 5:**
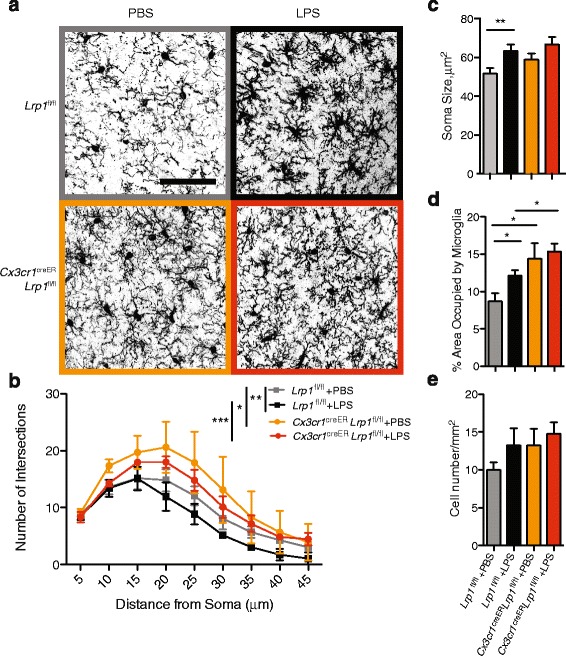
LRP1 deficient microglia have an amoeboid morphology. *Cx3cr1* ^creER^-*Lrp1* ^fl/fl^ and *Lrp1* ^fl/fl^ mice were injected daily with LPS (1 mg/kg) for 4 consecutive days. Brain sections were stained with Iba1 antibody, imaged by confocal microscopy and analyzed with ImageJ. **a** Representative images of microglia and **b** corresponding Sholl analyses. *p < 0.05; **p < 0.01; ***p < 0.001, 2-way ANOVA. (Scale bar = 60 μm). **c** Soma size of the microglia. 10 microglia per animal were quantified for each experimental group. **p < 0.01, Student’s t-test. **d** Quantification of area covered by Iba1+ microglia per image. *p < 0.05, Student’s t-test. **e** Numbers of microglia per unit area (*n* = 3–4 mice per condition). Values represent mean ± s.e.m

Since our results suggested that T cell and BBB function is normal in the absence of LRP1 expression in CX3CR1 positive cells, we hypothesized that the increased disease severity is due to an intrinsic function of LRP1 in microglia. Therefore, we analyzed the morphology of microglia in the cortex of *Cx3cr1*^creER^-*Lrp1*^fl/fl^ and control mice at both the resting state, and after inducing microgliosis via peripheral administration of LPS for 4 consecutive days, as described [37]. LRP1 status in microglia did not affect the weight of the animals injected with LPS (Additional file 1: Figure S4). We chose this model instead of EAE because of the lack of good markers to differentiate microglia from peripheral myeloid cells by immunochemistry. Because this model of neuroinflammation lacks recruitment of peripheral immune cells, a more focused analysis of microglia morphology is possible [37]. Brain sections were stained with Iba1 specific antibody and microglial ramifications were quantified by Sholl analysis in the cortex (Fig. 5a-b). In the healthy control mice, microglia appear thin and ramified (Fig. 5b, grey curve) [37]. Surprisingly, microglia lacking LRP1 expression appeared more amoeboid than the control cells at baseline, with a denser cell body and thickened proximal dendrites (Fig. 5b, grey vs. orange curves). Whereas LPS administration in control mice leads to the transition of microglial morphology into the bushy and thickened appearance (Fig. 5b, grey vs black curves), LPS had no effect on the appearance and branching of LRP1 deficient microglia (Fig. 5b, orange vs red curves). LPS administration significantly increased the soma size of control microglia, but not the microglia lacking LRP1 expression (Fig. 5c). Quantification of the area covered by Iba1+ cells confirms that LRP1 deletion leads to microglial hypertrophy. The morphology of these LRP1 deficient microglia are similar in appearance to the microglia observed in LPS treated control mice (Fig. 5d). These morphological changes were not associated with an overall change in numbers of microglia (Fig. 5e). Our results suggest that the removal of LRP1 alters microglial morphology in a manner similar to treatment with LPS, a potent neuroinflammatory stimulus.

### Microglia lacking LRP1 have a pro-inflammatory signature

**Fig. 6:**
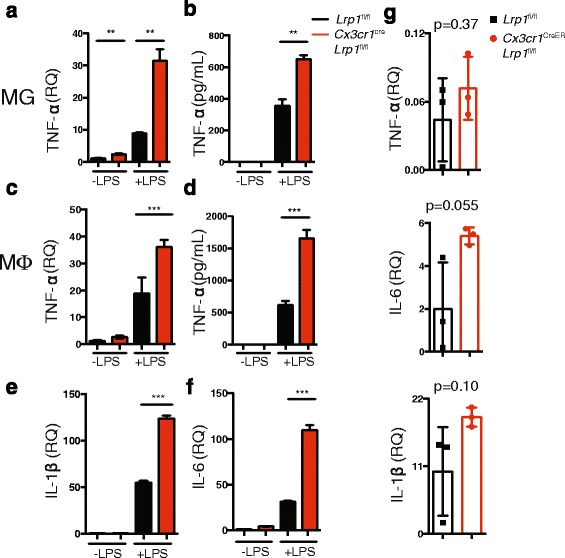
LRP1 deficient myeloid cells have a pro-inflammatory signature. Microglia (MG) and BMDM (MΦ) isolated from *Cx3cr1* ^cre^-*Lrp1* ^fl/fl^ and *Lrp1* ^fl/fl^ mice were treated with LPS (1 μg/mL) for 3 h (qPCR) or 24 h (ELISA). TNF-α expression in microglia was determined by qPCR (**a**) and ELISA (**b**). TNF-α expression in macrophages was determined by qPCR (**c**) and ELISA (**d**). Expression of IL-1β (**e**) and IL-6 (**f**) in macrophages was determined by qPCR. **g** Transcript expression of IL-1β, IL-6, and TNF-α from the CNS of *Cx3cr1* ^creER^-*Lrp1* ^fl/fl^ and *Lrp1* ^fl/fl^ mice at the peak of EAE. 5, **p < 0.01, ***p < 0.001; Student’s t-test

One potential mechanism by which microglia lacking LRP1 could affect EAE pathology is through increased production of inflammatory mediators, as LRP1 functions as an inhibitor of the inflammatory response [15]. To test the inflammatory response in LRP1 deficient microglia, we isolated primary microglia from *Lrp1*^fl/fl^ and *Cx3cr1*^cre^-*Lrp1*^fl/fl^ mice and analyzed their production of TNF-α, a key cytokine involved in EAE pathology [38]. LPS stimulation of LRP1 deficient microglia results in increased production of TNF-α at both the transcript (Fig. 6a) and the protein level (Fig. 6b). This increase in TNF-α is comparable to the one observed in primary cultures of BMDM (Fig. 6c-d), as we have previously reported [15]. In BMDM, the pro-inflammatory signature generated by the lack of LRP1 also extends to IL-1β and IL-6 production (Fig. 6e-f). More importantly, this pro-inflammatory signature was also observed in the CNS of *Cx3cr1*^creER^-*Lrp1*^fl/fl^ mice at the disease onset, as detected by qPCR (Fig. 6g). Taken together, our results demonstrate that, in the absence of LRP1, microglia adopt a pro-inflammatory phenotype that could contribute to increased pathology in EAE.

### LRP1 blocks NF-kB function

**Fig. 7:**
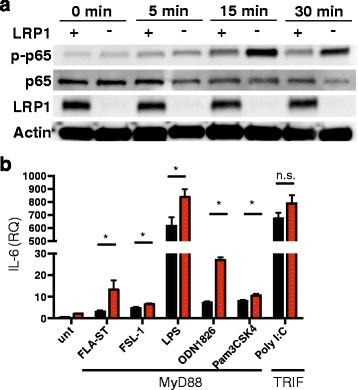
LRP1 is an inhibitor of NF-kB through the MyD88 pathway. **a** Macrophages isolated from *Cx3cr1* ^cre^-*Lrp1* ^fl/fl^ (LRP1-) and *Lrp1* ^fl/fl^ (LRP1+) mice were treated with LPS (1 μg/ml) and the expression of p-p65, p65, LRP1 and actin were determined by immunoblot. Representative of 3 independent experiments). **b** LRP1+ and LRP1- macrophages were treated with TLR ligands and expression of IL-6 was determined by qPCR analysis. *p < 0.05; Student’s t-test, mean ± s.e.m

Production of cytokines, like TNF-α, IL-6 and IL-1β, is tightly controlled via signaling through NF-kB, a transcriptional master regulator of the inflammatory response [39]. Indeed, recent studies have demonstrated that blocking NF-kB activation specifically in microglia is sufficient to offer protection during EAE [9]. Previous reports have highlighted the fact that LRP1 can act as an inhibitor of inflammation through regulation of NF-kB activity [15]. We therefore hypothesized that LRP1 may function as a regulator of NF-kB mediated inflammatory response in microglia. Because the inflammatory mediator production appears similar in cultured microglia and BMDM lacking LRP1 (Fig. 6), we began by treating *Lrp1*^fl/fl^ and *Cx3cr1*^cre^-*Lrp1*^fl/fl^ BMDM with LPS and collecting protein extracts at several time points after stimulation. Protein extracts were analyzed for the phosphorylation status of the NF-kB subunit p65. As is shown in Fig. 7a, LPS stimulated LRP1 deficient macrophages have increased levels of phosphorylated p65 (p-p65), in comparison to control cells. LPS is the only Toll-like receptor (TLR) ligand known to activate two different signaling pathways: the MyD88 and the TRIF pathway [39]. To elucidate which arm of the signaling cascade is regulated by LRP1, we treated BMDM with a panel of TLR ligands that are associated with the TRIF (TLR3 ligand: PolyI:C), or the MyD88 adaptor response (TLR1 and 2 ligand: Pam3CSK4; TLR5 ligand: FLA-ST; TLR6 ligand: FSL-1; TLR9 ligand: ODN1826) [40]. BMDM were treated for 3 h and IL-6 expression was determined by qPCR analysis. IL-6 expression was increased in LRP1 deficient macrophages when the MyD88 pathway was engaged by the activation of TLR 1, 2, 4, 5, 6, and 9. On the contrary, TLR3-mediated production of IL-6 was not affected by the status of LRP1, showing that LRP1 may inhibit the NF-kB signaling pathway in a MyD88, but not TRIF dependent manner (Fig. 7b). Together, these results suggest that LRP1 functions as a regulator of NF-kB activity through modulation of the MyD88 dependent arm of the inflammatory response. This function of LRP1 might explain why its removal in the microglial compartment is detrimental during CNS autoimmunity.

## Discussion

In this study, we explore the function of LRP1 expression in myeloid cells during MS using the mouse model EAE. To begin, we have made the novel observation that LRP1 protein expression is significantly increased in human MS lesions, compared to normal appearing brain tissue. The cellular compartments involved in the increase of LRP1 immunoreactivity included the myeloid cells and astrocytes. This observation is in agreement with our previous work showing that LRP1 expression is upregulated during EAE [11] and the work of Hendrickx et al., showing that LRP1 transcript is increased at the rim of MS lesions [41]. The LRP1 expression increase by glia is not limited to MS, as previous work has demonstrated a similar pattern during CNS injury and neoplasia [42]. Therefore, although our study centered on LRP1 function in myeloid cells, future studies will be needed to understand the role of LRP1 in astrocytes.

Here, we have explored the contribution of LRP1 expression during EAE in two distinct myeloid cell populations: Peripheral macrophages and microglia. To our surprise, we have discovered that LRP1 deletion in peripheral macrophages had no detectable impact on EAE progression. This result is in stark contrast with the consequence of LRP1 deletion in microglia, which leads to a significant worsening of disease progression. We, and others, have previously demonstrated that macrophages lacking LRP1 expression display a pro-inflammatory phenotype in vitro, characterized by increased production of inflammatory mediators, chemokines and an M1-type macrophage skewing [15, 43, 44], suggesting that the removal of LRP1 in macrophages could impact the progression of EAE in a detrimental manner. Therefore, we were surprised to find that LRP1 deletion in *LysM*^cre^-*Lrp1*^fl/fl^ mice did not result in increased disease severity in two separate EAE protocols (varying in the amount of mycobacterium used for the preparation of the complete Freund’s adjuvant). One potential explanation for these observations is that the peripheral immune cells are primed for a powerful inflammatory response prior to their entry into the CNS, and the removal of LRP1 does not alter their function in the diseased tissue. Future studies are necessary to distinguish whether LRP1 deletion in peripheral myeloid cells alters their function under homeostatic conditions.

On the contrary, ablation of LRP1 in microglia had a significant impact on EAE disease severity and progression. Microglia are the resident myeloid cells of the CNS and their function in EAE remains actively debated; while some studies suggest that they actively participate in disease initiation and peripheral immune cell recruitment [9, 45], others show that microglia remain relatively inert during EAE [8]. Microglia have also been reported to express higher levels of the LRP1 transcript, compared to the peripheral macrophages, in both EAE and the homeostatic conditions [46, 47]. Therefore, we propose that microglia lacking LRP1 acquire an inflammatory phenotype that leads to disease exacerbation in EAE. In agreement with this hypothesis are the observations that LRP1 deficient microglia appear amoeboid and hypertrophic, even in the absence of ongoing disease. The transition from a ramified morphology to a more compact appearance by microglia has been described by many groups as an hallmark of inflammation [37, 48]. The fact that microglia appear activated in the healthy brain does suggest that they are primed to produce a more robust inflammatory response. Indeed, microglia lacking LRP1 secrete more TNF-α after LPS stimulation in vitro, a key cytokine involved in EAE pathology [38].

On a mechanistic level, we propose that LRP1 is an inhibitor of NF-kB signaling in myeloid cells, in agreement with our previous studies [15, 44]. Here, for the first time, we show that LRP1 only influences the MyD88 arm of TLR signaling, suggesting that LRP1 only has an effect on the extracellular TLR ligands [40]. Further studies are needed to understand if the role of LRP1 as an NF-kB inhibitor intersects with LRP1 function in the removal of myelin debris. Indeed, the function of LRP1 in the removal of degraded myelin and dying cells could be critical in maintaining CNS homeostasis under normal physiological conditions, as well as in removing the degenerating oligodendrocytes during MS [10, 11]. Proper clearance of the myelin debris is critical for brain function, as the release of degenerated myelin could further propagate inflammation and damage the CNS. Furthermore, myelin-laden macrophages isolated from the brain are known to have decreased inflammatory responses, when compared to macrophages that have not engulfed myelin [49]. The receptors and mechanisms that initiate the anti-inflammatory response to apoptotic cell or myelin engulfment are still poorly understood [50]. Further studies will be needed to understand if LRP1 mediated signaling in microglia can initiate an anti-inflammatory response, thereby exerting protection in EAE and, perhaps, MS.

## Conclusion

In conclusion, we demonstrated that LRP1 expression is significantly upregulated by myeloid cells in active MS lesions. To study the role of LRP1 in myeloid cells, we induced EAE in mice lacking LRP1 in microglia or in macrophages and showed that only microglial LRP1 was protective, as animal lacking LRP1 in this compartment experienced a worse clinical outcome. At the mechanistic level, our study demonstrates that LRP1 regulates inflammation by inhibiting NF-kB, a master regulator of inflammation, via a MyD88 dependent pathway.

## Abbreviations

MS, Multiple sclerosis; CNS, central nervous system; LRP1, Low-density lipoprotein receptor-related protein-1; EAE, experimental autoimmune encephalomyelitis; MOG, myelin oligodendrocyte glycoprotein; BMDM, bone marrow derived macrophages; BBB, Blood Brain Barrier.

## Additional file

Additional file 1:
**Figure S1.** Higher dose of mycobacterium does not affect EAE progression in *LysM* ^*Cre*^ *-Lrp1* ^*fl/fl*^ and *Lrp1fl/fl* mice. **Figure S2.** CX3CR1 haplodeficiency does not affect EAE progression. EAE was induced in *Cx3cr1* ^*creER*^ *-Lrp1* ^*fl/fl*^ and *Lrp1* ^*fl/fl*^ mice without tamoxifen treatment. **Figure S3.** Flow cytometry gating strategy to identify macrophages, microglia and lymphocytes (Figs. 2 and 3). **Figure S4.** LRP1 deficiency in microglia does not alter weight loss after LPS treatment. *Cx3cr1* ^*creER*^ *-Lrp1* ^*fl/fl*^ and *Lrp1* ^*fl/fl*^ mice were injected with LPS (1 mg/kg) for 4 consecutive days and the weight of the animals was monitored daily. (PDF 1368 kb)

## References

[1] Compston A, Coles A (2008). Multiple sclerosis. Lancet.

[2] Ajami B (2011). Infiltrating monocytes trigger EAE progression, but do not contribute to the resident microglia pool. Nat Neurosci.

[3] Lucchinetti CF, Bruck W, Lassmann H (2004). Evidence for pathogenic heterogeneity in multiple sclerosis. Ann Neurol.

[4] Rinner WA (1995). Resident microglia and hematogenous macrophages as phagocytes in adoptively transferred experimental autoimmune encephalomyelitis: an investigation using rat radiation bone marrow chimeras. Glia.

[5] King IL, Dickendesher TL, Segal BM (2009). Circulating Ly-6C+ myeloid precursors migrate to the CNS and play a pathogenic role during autoimmune demyelinating disease. Blood.

[6] Nair A, Frederick TJ, Miller SD (2008). Astrocytes in multiple sclerosis: a product of their environment. Cell Mol Life Sci.

[7] Bogie JF, Stinissen P, Hendriks JJ (2014). Macrophage subsets and microglia in multiple sclerosis. Acta Neuropathol.

[8] Vainchtein ID (2014). In acute experimental autoimmune encephalomyelitis, infiltrating macrophages are immune activated, whereas microglia remain immune suppressed. Glia.

[9] Goldmann T (2013). A new type of microglia gene targeting shows TAK1 to be pivotal in CNS autoimmune inflammation. Nat Neurosci.

[10] Fernandez-Castaneda A (2013). Identification of the low density lipoprotein (LDL) receptor-related protein-1 interactome in central nervous system myelin suggests a role in the clearance of necrotic cell debris. J Biol Chem.

[11] Gaultier A (2009). Low-density lipoprotein receptor-related protein 1 is an essential receptor for myelin phagocytosis. J Cell Sci.

[12] Gardai SJ (2005). Cell-surface calreticulin initiates clearance of viable or apoptotic cells through trans-activation of LRP on the phagocyte. Cell.

[13] Lampron A (2015). Inefficient clearance of myelin debris by microglia impairs remyelinating processes. J Exp Med.

[14] Lillis AP (2008). LDL Receptor-Related Protein 1: Unique Tissue-Specific Functions Revealed by Selective Gene Knockout Studies. Physiol Rev.

[15] Gaultier A (2008). Regulation of tumor necrosis factor receptor-1 and the IKK-NF-kappaB pathway by LDL receptor-related protein explains the antiinflammatory activity of this receptor. Blood.

[16] van Loo G (2006). Inhibition of transcription factor NF-kappaB in the central nervous system ameliorates autoimmune encephalomyelitis in mice. Nat Immunol.

[17] Hilliard B (1999). Experimental autoimmune encephalomyelitis in NF-kappa B-deficient mice:roles of NF-kappa B in the activation and differentiation of autoreactive T cells. J Immunol.

[18] Hilliard BA (2002). Critical roles of c-Rel in autoimmune inflammation and helper T cell differentiation. J Clin Invest.

[19] Pahan K, Schmid M (2000). Activation of nuclear factor-kB in the spinal cord of experimental allergic encephalomyelitis. Neurosci Lett.

[20] Dasgupta S (2004). Antineuroinflammatory effect of NF-kappaB essential modifier-binding domain peptides in the adoptive transfer model of experimental allergic encephalomyelitis. J Immunol.

[21] Popescu BF, Pirko I, Lucchinetti CF (2013). Pathology of multiple sclerosis: where do we stand?. Continuum (Minneap Minn).

[22] Rohlmann A (1996). Sustained somatic gene inactivation by viral transfer of Cre recombinase. Nat Biotechnol.

[23] Clausen BE (1999). Conditional gene targeting in macrophages and granulocytes using LysMcre mice. Transgenic Res.

[24] Cronk JC (2015). Methyl-CpG Binding Protein 2 Regulates Microglia and Macrophage Gene Expression in Response to Inflammatory Stimuli. Immunity.

[25] Remick DG (2002). Six at six: interleukin-6 measured 6 h after the initiation of sepsis predicts mortality over 3 days. Shock.

[26] Wolf BB (1992). Characterization and immunohistochemical localization of alpha 2-macroglobulin receptor (low-density lipoprotein receptor-related protein) in human brain. Am J Pathol.

[27] Ginhoux F (2010). Fate mapping analysis reveals that adult microglia derive from primitive macrophages. Science.

[28] Cho IH (2008). Role of microglial IKKbeta in kainic acid-induced hippocampal neuronal cell death. Brain.

[29] Overton CD (2007). Deletion of macrophage LDL receptor-related protein increases atherogenesis in the mouse. Circ Res.

[30] Stromnes IM, Goverman JM (2006). Active induction of experimental allergic encephalomyelitis. Nat Protoc.

[31] Yona S (2013). Fate mapping reveals origins and dynamics of monocytes and tissue macrophages under homeostasis. Immunity.

[32] Subramanian M (2014). An AXL/LRP-1/RANBP9 complex mediates DC efferocytosis and antigen cross-presentation in vivo. J Clin Invest.

[33] Jung S (2000). Analysis of fractalkine receptor CX(3)CR1 function by targeted deletion and green fluorescent protein reporter gene insertion. Mol Cell Biol.

[34] Bennett J (2010). Blood-brain barrier disruption and enhanced vascular permeability in the multiple sclerosis model EAE. J Neuroimmunol.

[35] da Fonseca AC (2014). The impact of microglial activation on blood-brain barrier in brain diseases. Front Cell Neurosci.

[36] Ramirez SH (2012). Activation of cannabinoid receptor 2 attenuates leukocyte-endothelial cell interactions and blood-brain barrier dysfunction under inflammatory conditions. J Neurosci.

[37] Chen Z (2012). Lipopolysaccharide-induced microglial activation and neuroprotection against experimental brain injury is independent of hematogenous TLR4. J Neurosci.

[38] Selmaj KW, Raine CS (1995). Experimental autoimmune encephalomyelitis: immunotherapy with anti-tumor necrosis factor antibodies and soluble tumor necrosis factor receptors. Neurology.

[39] Lu YC, Yeh WC, Ohashi PS (2008). LPS/TLR4 signal transduction pathway. Cytokine.

[40] Yu L, Wang L, Chen S (2010). Endogenous toll-like receptor ligands and their biological significance. J Cell Mol Med.

[41] Hendrickx DA (2013). Selective upregulation of scavenger receptors in and around demyelinating areas in multiple sclerosis. J Neuropathol Exp Neurol.

[42] Lopes MB (1994). Expression of alpha 2-macroglobulin receptor/low density lipoprotein receptor-related protein is increased in reactive and neoplastic glial cells. FEBS Lett.

[43] May P, Bock HH, Nofer JR (2013). Low density receptor-related protein 1 (LRP1) promotes anti-inflammatory phenotype in murine macrophages. Cell Tissue Res.

[44] Staudt ND (2013). Myeloid cell receptor LRP1/CD91 regulates monocyte recruitment and angiogenesis in tumors. Cancer Res.

[45] D’Mello C, Le T, Swain MG (2009). Cerebral microglia recruit monocytes into the brain in response to tumor necrosis factoralpha signaling during peripheral organ inflammation. J Neurosci.

[46] Lewis ND (2014). RNA sequencing of microglia and monocyte-derived macrophages from mice with experimental autoimmune encephalomyelitis illustrates a changing phenotype with disease course. J Neuroimmunol.

[47] Butovsky O (2014). Identification of a unique TGF-beta-dependent molecular and functional signature in microglia. Nat Neurosci.

[48] Kozlowski C, Weimer RM (2012). An automated method to quantify microglia morphology and application to monitor activation state longitudinally in vivo. PLoS One.

[49] Boven LA (2006). Myelin-laden macrophages are anti-inflammatory, consistent with foam cells in multiple sclerosis. Brain.

[50] Henson PM, Bratton DL (2013). Antiinflammatory effects of apoptotic cells. J Clin Invest.

