# Supplementary material for: LRP1 expression in microglia is protective during CNS autoimmunity
Source: Acta Neuropathol Commun. 2016 Jul 11;4:68. doi: 10.1186/s40478-016-0343-2 (PMC4940960; doi:10.1186/s40478-016-0343-2)
Supplement: Additional file 1: — Figure S1. Higher dose of mycobacterium does not affect EAE progression in LysM Cre -Lrp1 fl/fl and Lrp1fl/fl mice. Figure S2. CX3CR1 haplodeficiency does not affect EAE progression. EAE was induced in Cx3cr1 creER -Lrp1 fl/fl and Lrp1 fl/fl mice without tamoxifen treatment. Figure S3. Flow cytometry gating strategy to identify macrophages, microglia and lymphocytes (Figs. 2 and 3). Figure S4. LRP1 deficiency in microglia does not alter weight loss after LPS treatment. Cx3cr1 creER -Lrp1 fl/fl and Lrp1 fl/fl mice were injected with LPS (1 mg/kg) for 4 consecutive days and the weight of the animals was monitored daily. (PDF 1368 kb) [file 40478_2016_343_MOESM1_ESM.pdf]

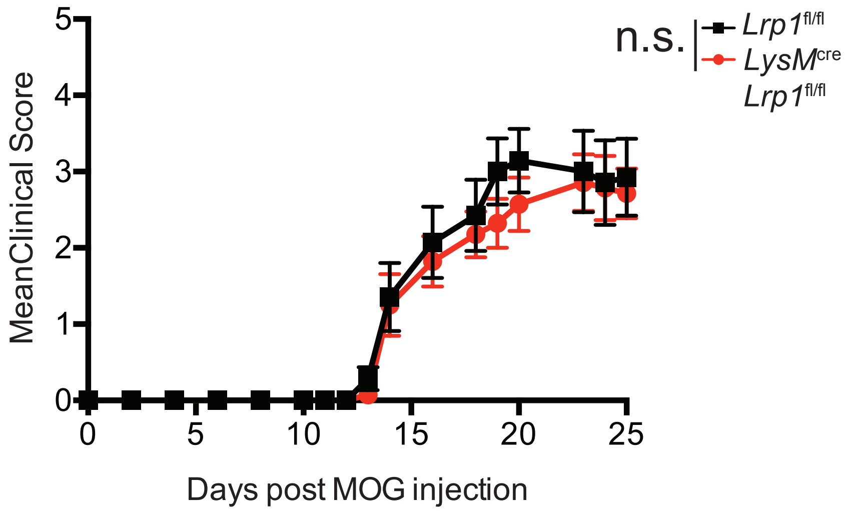

Supplementary Figure 1. Higher dose of mycobacterium does not affect EAE progression in *LysM-^{cre}-Lrp1$^{fl/fl}$* and *Lrp1$^{fl/fl}$* mice.

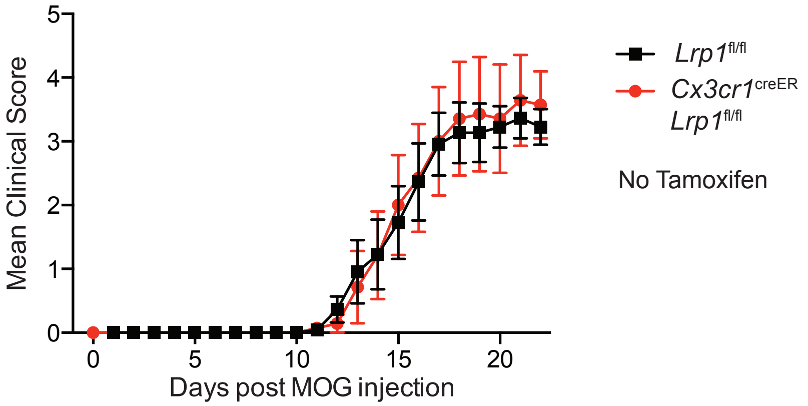

Supplementary Figure 2. CX3CR1 haplodeficiency does not affect EAE progression. EAE was induced in *Cx3cr1*^{creER}-*Lrp1*$^{fl/fl}$ and *Lrp1*$^{fl/fl}$ mice without tamoxifen treatment.

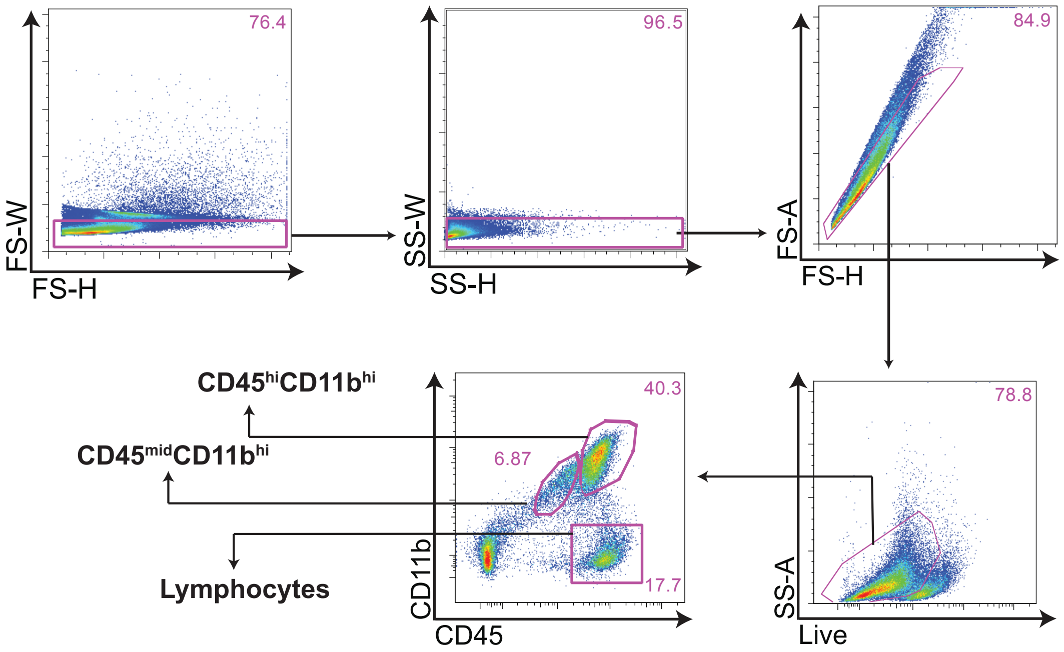

Supplementary Figure 3. Flow cytometry gating strategy to identify macrophages, microglia and lymphocytes (Figures 2 and 3).

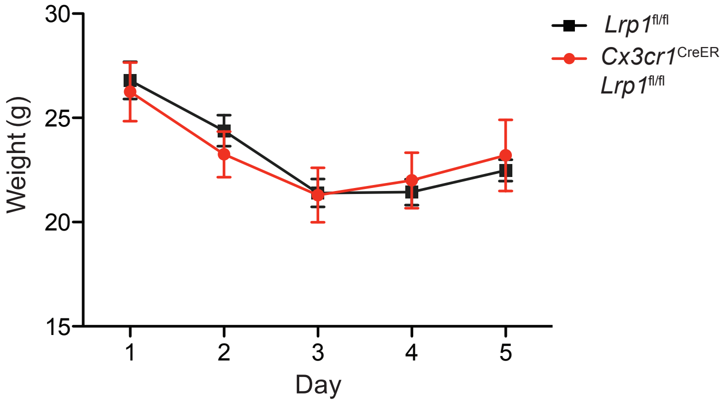

Supplementary Figure 4. LRP1 deficiency in microglia does not alter weight loss after LPS treatment. *Cx-3cr1^{creER}-Lrp1$^{fl/fl}$* and *Lrp1$^{fl/fl}$* mice were injected with LPS (1mg/kg) for 4 consecutive days and the weight of the animals was monitored daily.
